# Immune Checkpoint Inhibitor-Induced Limbic Encephalitis during Treatment with Atezolizumab in a Patient with Small-Cell Lung Cancer: A Case Report and Review of the Literature

**DOI:** 10.1155/2022/9290922

**Published:** 2022-01-06

**Authors:** Koki Nakashima, Yoshiki Demura, Kosuke Kurokawa, Toshihiro Takeda, Norihiro Jikuya, Masahiro Oi, Toshihiko Tada, Masaya Akai, Tamotsu Ishizuka

**Affiliations:** ^1^Depertment of Respiratory Medicine, Japanese Red Cross Fukui Hospital, 2-4-1, Tsukimi, Fukui-Shi, Fukui-Ken, Japan; ^2^Third Department of Internal Medicine, Faculty of Medical Sciences, University of Fukui, 23-3 Shimoaizuki, Eiheiji-Cho, Matsuoka-Gun, Fukui-Ken, Japan

## Abstract

Paraneoplastic neurological syndrome (PNS) is associated with malignancies, including small-cell lung cancer. Recently, PNS cases among patients with small-cell lung cancer (SCLC) induced by immune checkpoint inhibitors have increased. We herein report a 66-year-old man with SCLC who developed disorientation, dysphagia, and gait disturbance after three courses of treatment with atezolizumab. Brain magnetic resonance imaging revealed a high-intensity area in the bilateral temporal lobes. Blood test results were positive for anti-Hu and anti-Zic4 antibodies, which led to the diagnosis of limbic encephalitis as PNS. Some symptoms improved with intravenous administration of steroids and immunoglobulins.

## 1. Introduction 

Paraneoplastic neurological syndrome (PNS) is caused by an autoimmune process that develops in patients with any type of malignancy [1, 2]. Immune checkpoint inhibitors (ICIs) are effective treatment options for patients with malignancies, including small-cell lung cancer (SCLC) [3]. However, ICIs cause inflammatory side effects by increasing the activity of the immune system [4]. Therefore, ICIs are presumed to be a risk factor for PNS [5, 6]. In fact, cases of PNS induced by ICIs have recently increased [7–12].

Herein, we report a case of ICI-induced limbic encephalitis developed in a patient with SCLC. The present report suggests that clinicians should consider the possibility of PNS when patients develop neurological symptoms after ICI initiation.

## 2. Case Report

**Figure 1:**
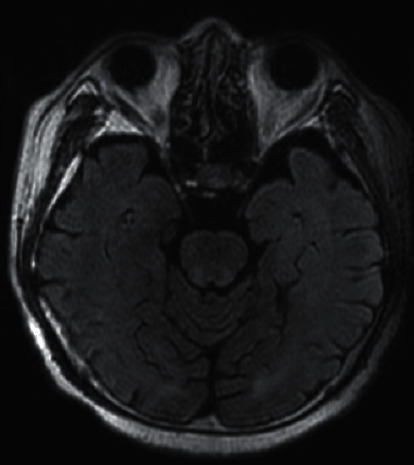
Fluid-attenuated inversion recovery (FLAIR) image of brain magnetic resonance imaging (MRI) before initiation of treatment with immune checkpoint inhibitor reveals no abnormal finding.

A 66-year-old man with a history of smoking for 40 years was referred to our hospital for abnormal chest radiograph findings. The patient had a history of bronchial asthma, with no history of autoimmune diseases. Computed tomography (CT) and positron emission tomography with 18F-fluorodeoxyglucose revealed a tumor mass in the right hilum, hilar and mediastinal lymph node swelling, and multiple lung metastases. Brain magnetic resonance imaging (MRI) showed no abnormal finding (Figure 1). Pathological findings of bronchoscopy of the primary tumor revealed SCLC. Therefore, the patient was diagnosed with extensive disease SCLC (ED-SCLC) and was treated with carboplatin and etoposide, and atezolizumab was initiated as first-line chemotherapy. Treatment led to a complete response.

**Figure 2:**
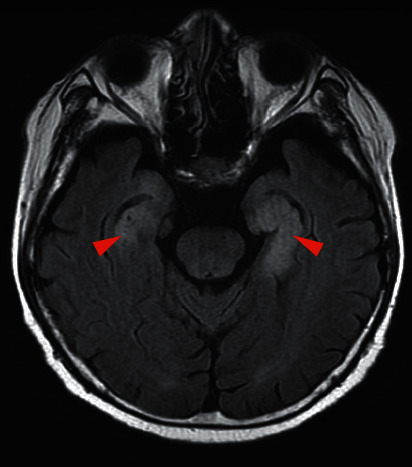
FLAIR image of brain MRI after development of neurological symptoms reveals high-intensity area in bilateral temporal lobes (red arrowheads).

**Figure 3:**
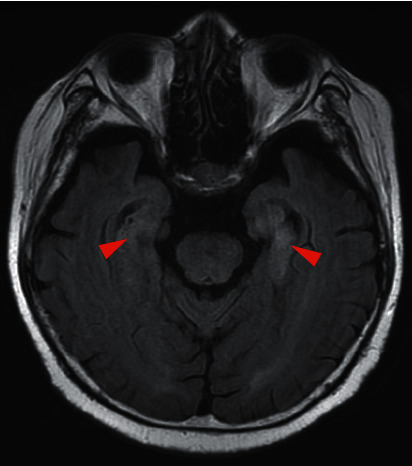
FLAIR image of brain MRI after development of neurological symptoms reveals slight improvement of high-intensity area in bilateral temporal lobes (red arrowheads).

The patient developed disorientation after three courses of chemotherapy over 2 months. Although follow-up without any treatment was continued, the disorientation worsened with coma. Dysphagia and gait disturbances due to muscle weakness also developed; however, we could not perform detailed neurological examination owing to the state of his consciousness. Fluid-attenuated inversion recovery (FLAIR) imaging of brain MRI after coma development showed a high-intensity area in the bilateral temporal lobes (Figure 2). Furthermore, anti-Hu and anti-Zic4 antibodies were highly detected in the blood test. The cerebrospinal fluid examination showed no evidence of tumor cells or infection, including herpes simplex virus and varicella-zoster virus (Table 1). Based on these results, anti-Hu and anti-Zic4 antibodies-positive limbic encephalitis as PNS was given as the final diagnosis. As steroid pulse therapy was initiated, the disturbance of consciousness improved. However, dysphagia and gait disturbance showed no improvement. Due to this, intravenous immunoglobulin (IVIG) therapy was also initiated leading to improvement of dysphagia, but not with gait disturbance. Brain MRI findings at 3 months after initiation of steroid treatment also improved slightly (Figure 3), and blood test at that time showed anti-Zic4 antibody negativity with anti-Hu antibody persistence.

At the time of writing, 6 months have passed since the development of limbic encephalitis, and the neurological symptoms did not worsen. Furthermore, a complete response was observed.

## 3. Discussion

In the present case, limbic encephalitis as PNS was diagnosed due to the following reasons. (1) Anti-Hu and anti-Zic4 antibodies were detected in the serum at the onset of neurological symptoms. (2) SCLC was presented at the onset of neurological symptoms. (3) SCLC is one of the most strongly associated tumors with PNS [7–12]. (4) MRI revealed a high-intensity area in the bilateral temporal lobes, which was consistent with limbic encephalitis. (5) No other possible cause was found for disorientation, such as central nervous system metastasis, stroke, or metabolic disorders in blood tests and brain MRI. (6) No evidence of meningeal carcinomatosis or infection in the cerebrospinal fluid was found.

Anti-Hu antibody is an auto-antibody associated with limbic encephalitis and sensory neuropathy [13]. Anti-Zic4 antibody is also associated with limbic encephalitis, cerebellar dysfunction, and sensory neuropathy [14]. The MRI findings of the patient were consistent with those of limbic encephalitis. Therefore, the neurological symptoms, disturbance of consciousness, dysphagia, and gait disturbance were considered to be caused by anti-Hu and anti-Zic4 antibodies-positive limbic encephalitis as PNS in the present case. Previous reports also showed several types of neurological symptoms of PNS in SCLC patients treated with ICIs (Table 2). However, a limitation of the present case is the non-examination of the neuronal cell surface antibodies. Neuronal cell surface antibody-mediated autoimmune encephalitis should be considered as a differential diagnosis [15]. A diagnosis of anti-Hu and anti-Zic4 antibodies-positive limbic encephalitis may be more likely, if neuronal cell surface antibodies, such as anti-n-methyl-D-aspartate (NMDA) receptor antibody, are fount to be negative.

ICI might have induced the neurological symptoms in the present case, since one of the mechanisms of immune-related adverse events is an increase in pre-existing autoantibodies [2, 12]. The neurological symptoms occurred 2 months after the first initiation of ICI, similar to previous reports (Table 2). Moreover, ICI-activated autoantibodies might be a reason why neurological symptoms were not improved completely. The persistence of neurological symptoms was also similar to previous reports (Table 2). ICI-induced PNS cases may be relatively rare in non-small-cell lung cancer patients, with only a few cases reported previously [16, 17]. Clinicians should consider the possibility of PNS especially when patients with SCLC complain of neurological symptoms after initiation of ICIs, especially after several months.

There is no established treatment for most cases of PNS. However, corticosteroids and other immunosuppressive drugs, such as cyclophosphamide or tacrolimus, IVIG, and plasma exchange, are often used in clinical practice [1]. In the present case, methylprednisolone and IVIG were used to treat PNS, and the disturbance of consciousness and dysphagia improved. However, gait disturbance did not improve. In previous cases, methylprednisolone and IVIG were the most frequently used treatments for PNS, but only a few patients had improved neurological symptoms completely (Table 2). Information about the appropriate treatments for PNS is needed in the future.

There are some limitations of present case. First, the examination of the neuronal cell surface antibodies was not performed. Second, other causes of limbic encephalitis, such as primary autoimmune encephalitis or iatrogenic encephalopathy, could not be ruled out completely.

In conclusion, we described a case of ICI-induced limbic encephalitis as PNS and reviewed the literature on cases of PNS induced by ICI in SCLC patients. Clinicians should consider the possibility of PNS when patients with SCLC develop neurological symptoms after ICI initiation.

## Figures and Tables

**Table 1:** Laboratory findings at the onset of PNS.

Anti-neuronal antibodies		Cerebrospinal fluid		
Amphiphysin	Negative	Appearance	Clear	
CV2	Negative	Cell count	5	/*μ*l
PNMA2	Negative	Poly	0	%
Ri	Negative	Mono	100	%
Yo	Negative	Protein	94	mg/dl
Hu	3+	Glucose	72	mg/dl
Recoverin	Negative	ADA	≦1	U/l
SOX1	Negative	HSV-PCR	Negative	
Titin	Negative	VZV-PCR	Negative	
Zic4	3+			
GAD65	Negative	Cytology	Class I	
Tr	Negative	Culture	Negative	

ADA, adenosine deaminase; HSV, herpes simplex virus; VZV, varicella-zoster virus.

**Table 2:** Details of cases of PNS induced by ICI in SCLC patients.

No.	Age	Sex	Country	ICI	Antibody	PNS	PNS symptoms	Time to onset	Treatment for PNS	Outcome	References
1	76	M	Japan	Atezolizumab	CRMP5	Striatal encephalitis	Forgetfulnes, irritability	5 months	Methylprednisolone	Improved	[7]

2	70	M	Japan	Atezolizumab	Hu, SOX1	Sensory polyneuropathy	Tactile and pain disturbances	1 month	IVIG	Not improved	[8]

3	66	F	China	Sintilimab	Hu	Encephalitis	Focal seizures	1.5 months	Methylprednisolone	Improved	[9]

4	62	F	USA	Nivolumab	Hu	Sensory polyneuropathy	Numbness in hands and feet, tremor, loss of dexterity, gait ataxia	a few days	Methylprednisolone, IVIG	Not improved	[10]

5	71	F	Switzerland	Nivolumab + ipilimumab	Hu	Limbic encephalitis	Short-term memory deficits	4 days	Methylprednisolone, natalizumab	Improved (not completely)	[11]

6	46	M	France	Pembrolizumab	Hu	Sensory neuropathy	Painful paresthesia, gait disturbance	3 months	Methylprednisolone, IVIG	Improved (temporary)	[12]

7	71	M	France	Atezolizumab	Hu	Encephalitis	Dizziness, vomiting, diplopia, gait disturbance	2 months	IVIG	Improved (not completely)	[12]

Present case	66	M	Japan	Atezolizumab	Hu, Zic4	Limbic encephalitis	Disorientation, dysphagia, gait disturbance	2 months	Methylprednisolone, IVIG	Improved (not completely)	

PNS, paraneoplastic neurological syndrome; ICI, immune checkpoint inhibitor; SCLC, small-cell lung cancer.
